# Advances in Biomarkers for CAR-T Treatment Resistance in Diffuse Large B-Cell Lymphoma

**DOI:** 10.2174/0113895575404134251003065725

**Published:** 2026-05-15

**Authors:** Si Qi Yi, Lu Lu Chen, Li Xv, Qin Jiao Fu, Yan Zhu Liu, YingYing Zhang, Meng Xian Jia, Xiang Min Tong, Ying Wang, Juan Qing Yue

**Affiliations:** 1 School of Pharmacy, Hangzhou Normal University, Hangzhou, Zhejiang, 311121, China;; 2 Hangzhou First People's Hospital, Affiliated Westlake University, Hangzhou, Zhejiang, 310006, China;; 3 School of Pharmacy, Zhejiang Chinese Medical University, Hangzhou, Zhejiang, 310053, China;; 4 Department of Orthopedics (Spine Surgery), the First Affiliated Hospital of Wenzhou Medical University, Wenzhou, Zhejiang, China

**Keywords:** CAR-T, diffuse large B-cell lymphoma, drug resistance, biomarkers, cell therapy, doxorubicin

## Abstract

Chimeric antigen receptor (CAR)-T cell therapy has made significant progress in treating hematologic tumors, particularly diffuse large B-cell lymphoma (DLBCL). However, over 50% of patients are resistant after CAR-T cell therapy for DLBCL, and drug resistance is gradually becoming a major obstacle limiting its efficacy. This review primarily focuses on the mechanisms of resistance to CAR-T cell therapies, including immune escape of tumor cells, CAR-T cell dysfunction, and the tumor microenvironment (TME). It explores potential predictive markers of resistance in the context of relevant resistance mechanisms, such as tumor cell surface antigens, CAR-T cell function, and gene mutation-related substances within the tumor microenvironment. The exploration of these potential predictive markers yields some relevant insights for future studies on resistance to CAR-T in the treatment of DLBCL.

## 1 INTRODUCTION

### 1.1 Diffuse Large B-Cell Lymphoma: Current Status

Diffuse large B-cell lymphoma (DLBCL) is a prevalent subtype of non-Hodgkin lymphoma (NHL), accounting for approximately 30% of all NHL cases in Western countries and more than 40% of all NHL cases in Asian countries [1]. The global incidence of DLBCL is increasing, with approximately 150,000 new cases being diagnosed annually. This number represents nearly 30% of all non-Hodgkin lymphoma cases worldwide each year [2]. Although 60 to 70 % of the patients can be treated with current first-line standards of care, the majority of the remaining patients develop treatment resistance and poorer clinical outcomes, posing significant challenges to the quality of life and outcomes for patients [3].

### 1.2 Current Treatment of DLBCL

In recent years, with the growing understanding of DLBCL and the success of clinical trials, great progress has been made in the treatment of DLBCL. The standard first-line therapy for newly diagnosed DLBCL patients is the R-CHOP regimen, which comprises rituximab combined with cyclophosphamide, doxorubicin, vincristine, and prednisone [4]. The majority of patients experience a favorable response, with over 50% achieving remission. However, a significant proportion, ranging between 30 to 40%, encounter a relapse or become unresponsive to the standard treatment. This remains a significant cause of death and disease rates due to the scarcity of treatment strategies [5]. Notably, the FDA has approved polatuzumab vedotin in combination with rituximab, cyclophosphamide, doxorubicin, and prednisone (Pola-R-CHP) for first-line treatment. Recent findings have demonstrated a promising clinical benefit with Pola-R-CHP in previously untreated patients with intermediate or high-risk DLBCL [6]. For patients with primary refractory or relapsed (R/R) DLBCL, therapeutic strategies require individualized approaches based on clinical profiles. Based on the PARMA study, salvage chemotherapy followed by high-dose chemotherapy with autologous stem cell transplantation (HDC/ASCT) has been the standard second-line therapy for R/R DLBCL over the past two decades [7]. Currently, CAR-T cell therapy has emerged as the preferred second-line treatment for R/R DLBCL [8]. In third-line and subsequent therapies, CAR-T remains the cornerstone intervention [4]. Concurrently, bispecific antibodies (such as epcoritamab and glofitamab) serve as critical "off-the-shelf" immunotherapeutic alternatives for patients who are ineligible for or have failed prior CAR-T therapy [9]. Among the most promising in contemporary oncological therapies is chimeric antigen receptor (CAR) T-cell therapy, showcasing impressive effectiveness across various blood cancers, especially in cases of DLBCL [10].

### 1.3 Promising CAR-T Cell Therapy and Application of DLBCL

In the treatment of certain blood system tumors, such as DLBCL and acute lymphoblastic leukemia (ALL), CAR-T cells are emerging as a new option [11, 12]. It is an immunotherapy that utilizes the patient's own T cells, customized to their specific needs. CAR (chimeric antigen receptor) is a synthetic receptor capable of binding to MHC-independent target antigens and directing cytotoxic immune cells toward those that express the antigen in a new way. A CAR is composed of three structural domains: an extracellular antigen recognition domain, an intracellular signal transduction domain, and a transmembrane domain. A single-chain variable fragment (scFv) is used to specifically bind to tumor surface antigens within the extracellular domain. Tumor antigens are generally categorized into tumor-associated antigens (TAAs) and tumor-specific antigens (TSAs), with TAAs being the more prevalent group. When the scFv recognizes and binds to a TAA, it triggers the activation of the CAR-T cell, which then transmits an activation signal to the intracellular domain [13]. The primary role is to substitute the TCR-CD3 complex and convey the signals necessary for T-cell activation [14]. In CAR-T cell therapy, T cells of patients are extracted, genetically modified to express CAR, and then reinjected back into the body to allow them to recognize and kill cancer cells. They can also remain in the body for extended periods, providing a prolonged anti-tumor response [15]. In addition to using healthy donor cells, allogeneic CAR-T cells can also be used for transformation therapy, which has several potential advantages compared to conventional methods. These include immediate availability of CAR-T cells for patient treatment, standardization of CAR-T cell products, the ability to reengineer or combine CAR-T cells to target different antigens, and cost reduction through industrialized processes. As technology evolves, future CAR-T cell therapies are likely to undergo continuous improvement and innovation [16].

CAR-T cell therapy in DLBCL has made remarkable breakthroughs, and numerous reports based on real-world data worldwide have confirmed the high response rate, longer response duration, and improved survival outcomes associated with CAR-T in DLBCL [17]. Currently, two CAR-T cell therapies have been approved by the FDA for the treatment of DLBCL: axicabtagene ciloleucel (commercial name Yescarta) and tisagenlecleucel (commercial name Kymriah) [17, 18]. Axicabtagene ciloleucel is a CAR-T cell therapy targeting the CD19 antigen. In a clinical trial, a total of 111 patients were enrolled, among whom 101 had relapsed or refractory (R/R) large B-cell lymphoma (LBCL) after at least two prior lines of therapy and received a single infusion of axi-cel. An overall response rate (ORR) of 82% and a complete remission rate (CR) of 54% were achieved [19-22]. These studies suggested that CAR-T cell therapy can provide significant clinical benefit in some patients with advanced or refractory DLBCL [20]. Another example is tisagenlecleucel, which is also a CAR-T cell therapy targeting the CD19 antigen. In the JULIET trial, the ORR of patients treated with stilbestrol (Kymriah) was 52%, and the complete remission (CR) rate was 41% [22].

Despite the promising future for CAR-T in treating DLBCL, the development of treatment resistance remains a common and recurring challenge. Studies have reported that approximately 40-50% of patients with DLBCL develop primary or secondary resistance [20, 23, 24]. This means that almost half of the people treated will experience resistance. Moreover, such relapses occur within 12 months of infusion (a total recurrence rate of 61% within the first 12 months after CAR-T infusion) or after the first year (a recurrent comorbidity rate of 24%) [25]. Drug resistance indicates that CAR-T cells are ineffective in controlling the growth of tumor cells, which can lead to rapid disease progression. Some patients with CAR-T resistance will relapse and progress again, and there is no established standard treatment protocol for this. Most patients are treated with immune checkpoint inhibitors, lenalidomide, chemotherapy, radiation, or a combination of these therapies [26]. Overall, fewer than 25% of DLBCL patients who experienced progression after CD19 CAR-T therapy showed a positive response to subsequent treatments. Additionally, the median overall survival (OS) for these patients was 3.6 months [27, 28].

This review discusses the currently known mechanisms of resistance to CAR-T therapy, potential predictive biomarkers of resistance, and strategies to overcome resistance in CAR-T treatment of DLBCL. A systematic search was conducted in PubMed, Embase, Web of Science, and Cochrane Library. The search strategy was developed based on the PICO framework (Population, Intervention, Comparison, and Outcome), using the following keywords (example for PubMed): ("diffuse large B-cell lymphomas" OR "DLBCL") AND ("CAR-T") AND ("resistance"). Medical Subject Headings (MeSH) were applied in PubMed. No language restrictions were initially imposed, but non-English articles were excluded during the screening process.

## 2 MECHANISMS OF RESISTANCE TO CAR-T THERAPY

### 2.1 Tumor Microenvironment (TME)

Typically, the TME refers to the tumor microenvironment, which comprises tumor cells, immune cells, stromal cells, extracellular matrix, and secreted factors [29]. It plays a central role in the genesis and progression of tumors, which not only provides cancer cells with a basic survival environment but also promotes their growth, invasion, and metastasis through the interaction between immune cells and cancer cells [30]. Immunosuppressive factors, metabolic competition, and physical barriers in TME may negatively affect the function and efficacy of CAR-T cells, leading to the development of drug resistance [31].

In tumor tissues, numerous immunosuppressive factors are present, including immune checkpoint molecules, regulatory T cells (Tregs), and myeloid-derived suppressor cells (MDSCs), which can inhibit the immune response and limit the activity of T cells, thus attenuating the killing effect of CAR-T cells [32]. For example, Treg cells, a substantial number of pro-tumor immune cells present in the TME, express Foxp3, and they act as defenders in maintaining immune homeostasis and peripheral tolerance, suppressing excessive autoimmunity, and potentially inhibiting the killing effect on tumor cells. The function of Treg cells is closely related to changes in the TME environment [33]. Many tumor diseases are usually caused by genetic mutations that have profound effects on cell differentiation, proliferation, survival, and genomic stability. A study explored the interactions between tumor cells and CAR-T cells, revealing that these interactions are related to higher mutations in the distinctive composition of the microenvironment, such as the TNFRSF14 mutation, suggesting that such alterations may enhance immune suppression [34].

TME metabolic factors play an important role in resistance to CAR-T cell therapy. Tumor cells alter their metabolic patterns to adapt to the need for rapid proliferation and survival, leading to significant alterations in the metabolic environment of the TME, which in turn affects the survival and function of CAR-T cells [35]. In TME, metabolic factors include a hypoxic environment, lack of nutrients, or acidic environment, all of which affect the activity and efficacy of CAR-T cells [36]. First, the tumor microenvironment is frequently low in oxygen, which decreases the metabolic activity and anti-tumor effect of CAR-T cells. In a hypoxic environment, CAR-T cells may secrete fewer effectors, increase apoptosis, and reduce their killing capacity through mechanisms that include upregulating the expression of PD-L1 on tumor cells and myeloid-derived suppressor cells (MDSCs). This upregulation impedes T-cell function, which subsequently contributes to the development of drug resistance [37]. Additionally, nutritional deprivation in TME can also affect CAR-T cell efficacy. In cancer, tumor cells must not only meet their energy demands but also support macromolecular biosynthesis (such as proteins, nucleotides, and lipids) and maintain redox homeostasis [38]. These proliferating cells require various nutrients, so they typically compete with CAR-T cells for essential nutrients, such as glucose and amino acids, which may lead to the inhibition of CAR-T cells without adequate nutritional support.

Physical barriers in the TME can significantly limit the penetration and mobility of CAR-T cells, thereby affecting the therapeutic efficacy of CAR-T. The extracellular matrix (ECM) in the tumor microenvironment (TME) can become particularly dense and fibrotic during tumor progression. Tumor-associated fibroblasts (CAFs), which are a major component of the tumor stroma, promote ECM deposition. The interaction between CAFs and ECM components further contributes to the formation of a physical barrier that impedes the infiltration of CAR-T cells into the tumor and limits their ability to eliminate cancer cells [39, 40]. For instance, recent research has revealed that in stereotypical lymphoma microenvironment archetype profiles, a predominant pathological feature is the abundance of cancer-associated fibroblasts (CAFs), driven by TGF-β signaling [41].

In conclusion, the complex tumor microenvironment poses a significant challenge to CAR-T resistance.

### 2.2 Defective CAR-T Cell Function

Defective CAR-T cell function refers to a condition in which CAR-T cells are impaired but not completely absent, and as a result, they are unable to effectively kill cancer cells. This may involve the activity and persistence of CAR-T cells [42]. Maintaining an optimal activation state of T cells is crucial for their proliferation and tumor killing. However, in contrast to effector and memory T cells, depleted T cells display several distinct characteristics. These differences include altered functions of effectors, such as decreased cytokine production and increased chemokine expression. Additionally, in depleted T cells, coinhibitory receptors (like PD-1, TIM-3, and TIGIT) are persistently expressed at high levels. They also have a reduced capacity for proliferation upon stimulation, exhibit changes in transcriptional programming involving the transcription factor TOX, and possess a unique epigenetic profile [43]. T cells require antigenic stimulation to be activated and expanded, but over-activation and excessive affinity for the target may lead to T-cell depletion and reduced killing activity, affecting the persistence of CAR-T cells and potentially leading to activation-induced cell death [44]. Modified CAR-T cells are engineered to augment their affinity for antigens and their capacity to respond to TCR and co-stimulatory signals. As a consequence of these enhancements, CAR-T cells typically exhibit continuous signaling, and their differentiation profiles are analogous or indistinguishable from those of exhausted T cells. It is possible, however, that interruptions in antigenic stimulation may enhance CAR-T cell function, potentially counteracting the established stability of T cell depletion and suggesting that the mechanisms of normal and CAR-T cell depletion may differ [45]. Research reported that the c-Jun AP1 transcription factor reduces T cell depletion when overexpressed [46]. Co-stimulatory structural domains play a significant role in the persistence and efficacy of CAR-T cells, and modification of the CAR structure improves the *in vivo* anti-tumor effect of CAR-T cell therapy by enhancing the maintenance of a memory immune response [47]. CAR-T cells incorporating 4-1BB domains have been shown to improve T-cell activity more effectively than those that utilize the CD28 structural domain [42]. During the interaction between CAR-T cells and tumor cells, the tumor cells may secrete immunosuppressive factors, including transforming growth factor β (TGF-β), interleukin-10 (IL-10), and PD-L1. These immunosuppressive factors can inhibit the activation and function of immune cells, including the CAR-T cells themselves. For example, the immunosuppressive factor PD-L1 binds to immune checkpoints on the surface of CAR-T cells, suppressing their activity [48, 49]. Tumor cells can also evade CAR-T cells by activating immunosuppressive pathways [48]. The activity of suppressor immune cells can be upregulated, such as regulatory T cells and tumor-associated macrophages, thereby inhibiting CAR-T cell function [50]. Additionally, a study reported that impaired death receptor signaling in tumor cells led to the failure of CAR-T-19 cytotoxicity and contributed to CAR-T-19 dysfunction. This suggests that alterations in signaling pathways can also drive CAR-T resistance [51]. Gene expression and epigenetic alterations associated with CAR-T cell depletion include the activation of nuclear factor of activated T-cells (NFAT) and the thymic high mobility group protein (TOX)-NR4A axis, which drives the T-cell depletion program and promotes the development of programmed cell death 1 (PDL), lymphocyte activation gene 3 (LAG3), T-cell immunoglobulin structural domains, and mucin structural domain 3 (TTM3), several inhibitory receptors (IRs), such as cytotoxic T lymphocyte antigen 4 (CTLA4). The therapeutic efficacy of CAR-T cell therapy is directly related to the quality of the cells. The process of CAR-T cell requires a large number of healthy T cells to be obtained from the patient. Patients with low-quality T cells have reduced therapeutic effectiveness [52, 53], and when they receive chemotherapy, it can result in lymphopenia and poor quality of CAR-T cell product. In addition, due to the reduced immune system function caused by human aging, studies have demonstrated that CAR-T cells generated from young donors exhibit higher transduction efficiency, enhanced cellular proliferation, increased signal activation, and greater tumor-killing capacity compared to those derived from older donors. Patients with advanced disease may also not be able to provide high-quality T cells for manufacturing CAR-T cells.

### 2.3 Antigen Escape

The primary mechanism of resistance to CAR-T cell therapy is antigen escape, where CAR-T cells are activated and kill cancer cells when they encounter a sufficient amount of antigen on the cancer cells' surfaces. Antigen escape occurs when tumor cells evade recognition and attack by the immune system by altering the expression of their surface antigens or through other mechanisms [54]. Antigen escape primarily involves antigen mutation, down-regulation, or loss, all of which reduce the effectiveness of CAR-T cell therapy and can lead to tumor recurrence [55]. Antigenic mutation is also a crucial aspect that contributes to antigenic escape. Gene mutations, gene rearrangements, and insertions or deletions in tumor cells may lead to structural changes in antigens, making it difficult for CAR-T cells to bind or recognize them effectively. In a recent study, mutations were found in exon 3 of CD19 in both relapsed and resistant patients treated with CAR-T cell therapy. These findings suggest that point mutations can promote immune escape from CAR-T cell therapy [56]. This finding highlights the importance of monitoring antigenic mutations and the need to identify markers associated with these mutations. In addition, downregulation of the antigen is one of the key mechanisms that lead to antigen escape. Data suggest that approximately 30% of recurrences of LBCL are CD19-negative, and another 30% exhibit CD19 expression levels below the antigen density threshold for commercial CARs [57]. For CAR-T cells to function effectively, a high antigen density is required, which allows a rapid selection of antigen loss or low antigenic variation [58-60]; however, tumor cells can cause reduced cell surface expression or reduced antigen density, which is sufficient to evade CAR-T cells, leading to the development of drug resistance [61]. Antigen loss is often caused by mutations affecting the antigen. This is mainly achieved through gene mutation or deletion, changing the expression of the relevant antigen. The expression of antigens is altered through a mechanism of epigenetic regulation, leading to deletion, which allows tumor cells to evade detection and attack by the immune system [62]. According to a study conducted by Sotillo and colleagues on patients who relapsed following CAR-T cell therapy targeting CD19, the molecular events were found to be correlated with the apparent deletion of CD19 expression. The study found that the underlying mechanism was the selection of pre-existing, selectively spliced CD19 isoforms with compromised CAR-T-19 epitopes [63]. This suggests that CD19 gene deletions and mutations may lead to loss of the CD19 antigen on tumor cells, resulting in CD19-negative recurrence. Additionally, immune escape mechanisms have been proposed as key contributors to antigen escape. Previous studies have suggested that combining immune checkpoint inhibitors to block PD-1 may enhance the efficacy of CAR-T in R/R DLBCL, particularly in patients with TP53 mutations [64]. These findings suggest that the combined use of CAR-T cell therapy and immune checkpoint inhibitors may be an effective strategy to overcome immune escape. However, this combination approach was evaluated in clinical trials involving patients with LBCL refractory to bridging therapy, demonstrating a favorable safety profile but no significant improvement in efficacy [65]. Therefore, careful consideration of the timing of administration is crucial during combination therapy with immune checkpoint inhibitors.

## 3 PREDICTIVE MARKERS OF CAR-T RESISTANCE

As resistance to CAR-T therapy has emerged as a significant challenge, exploring predictive markers of resistance is crucial for personalized treatment and the optimization of patient outcomes. Additionally, identifying predictive markers of CAR-T treatment resistance can help physicians predict the outcome of a patient's treatment and make individualized treatment choices, such as combination therapy, targeted therapy, or other immunotherapy methods. During the course of treatment, monitoring changes in resistance markers can detect signs of resistance in advance and take corresponding measures to prevent the further development of drug resistance.

### 3.1 Tumor Cell Surface Markers

The tumor cells in DLBCL originate from the B-cell lineage and usually express pan-B-cell antigens, such as CD19, CD20, and CD22 [66]. CAR-T therapy is currently targeting these three surface antigenic markers in clinical trials for DLBCL, and antigen loss is a key mechanism in the resistance to CAR-T therapy. A further understanding of drug resistance can be obtained by examining and testing for surface antigens.

#### 3.1.1 CD19

In CAR-T cell therapy, resistance in LBCL can be predicted by assessing changes in the expression of a marker indicated by tumor cells. CD19 is a highly significant target in CAR-T therapy. CD19 is a B-cell-specific cell surface marker that is widely expressed in the B-cell lineage, including both normal B cells and malignant cells in large B-cell lymphoma [67]. Targeted deletion, exemplified by CD19, is a well-documented mechanism of resistance. This phenomenon occurs when tumor cells develop CD19 exon splice variants or gene mutations, resulting in either a loss or modification of CD19 expression [68]. Related studies have reported CD19 loss in DLBCL after CD19-CAR-T treatment [69-71]. Therefore, in CAR-T therapy for DLBCL, drug resistance can potentially be predicted by detecting changes in the surface marker CD19.

#### 3.1.2 CD22

CD22 is also an antigen widely expressed on B-lymphocytes and is another potential therapeutic target for patients with B-cell malignancies [72]. The expression level of CD22 antigen in DLBCL patients has been found to correlate with resistance to specific treatments in several studies. There is some evidence that patients with hematological malignancies exhibiting high CD22 expression may respond better to CD22-directed CAR-T cell therapy [73, 74]. High CD22 expression may imply that more antigenic targets are available for CAR-T cells to target, thereby increasing the efficacy of the therapy. Downregulation of CD22 is one of the important mechanisms of resistance to CAR-T therapy [75, 76]. Resistance related to reduced antigen density has been demonstrated in clinical trials of CD22-directed CAR-T cells in patients with B-ALL [77]. B-ALL and DLBCL are distinct hematologic malignancies for which CAR-T therapies have been approved by the FDA [69, 78]. Although the role of CD22 in CAR-T resistance in DLBCL has not been fully investigated, future studies are expected to provide evidence supporting CD22 as a potential predictive marker of drug resistance in CAR-T treatment for DLBCL.

#### 3.1.3 CD20

CD20 is widely expressed in DLBCL cells, making it an important target for CAR-T therapy in the treatment of DLBCL [79]. The expression level of CD20 in CAR-T cells for the treatment of DLBCL may be correlated with the response to treatment [80, 81], suggesting that CD20 serves as an indicator of treatment response. Meanwhile, it has also been demonstrated that CD20 loss occurs in DLBCL following CAR-T treatment [70]. In a recent study, CD20 expression deficiency was also shown to be a characteristic of drug resistance mechanisms in DLBCL patients [82, 83]. Clinical studies have been conducted with bispecific anti-CD20 and anti-CD19 CAR-T cells in relapsed/refractory B-cell malignancies, which have been found to improve clinical response by attenuating the downregulation of target antigens as a mechanism of relapse [84, 85]. In summary, CD20 exhibits high potential as a predictive marker for future studies of CAR-T resistance in DLBCL.

### 3.2 T-Cell Function-Related Markers

#### 3.2.1 Acquired Drug Resistance

After CAR-T cell therapy, factors associated with T-cell function help predict drug resistance in patients with LBCL. There has been a negative correlation between T-cell depletion and response to the therapy. Elevated levels of immune checkpoint molecules, such as PD-1, TIM3, and LAG3, which indicate T-cell exhaustion, can destabilize immune synapses and suppress effective immune responses [86-87]. This can result in drug resistance or relapse following CAR-T cell therapy [88]. As a result of studies, T-cell function and efficacy are suppressed in the immunosuppressive microenvironment through the PD-1/PD-L1 axis, which is a mechanism of resistance to CAR-T therapy [87, 89, 90]. Blocking PD-1/PD-L1 allows for the reactivation of T-cell and effector functions, enabling them to kill tumor cells and benefit the patient [91]. Based on a study by Elise A et al, blocking PD-1 in patients with DLBCL tumors improved efficacy in CAR-T cell therapy [92]. In the future, it may be possible to monitor PD-1 expression to determine whether resistance develops during treatment. Additionally, IL-7 (interleukin-7), which is associated with lymphocyte depletion, serves as a prognostic marker in CAR-T therapy. Overexpression of IL-7 in CAR-T cells is required for the formation of T-cell compartments in lymphoid organs. Previous reports have shown that IL-7 signaling prevents or reverses T-cell depletion through multiple mechanisms [93-96]. A previous study also reported that in DLBCL patients treated with CD19 CAR-T cells, a cytokine profile characterized by high levels of serum monocyte chemoattractant protein-1 (MCP-1) and IL-7 was independently associated with a better response, making this immune profile a potential predictive biomarker [97]. As a correlate of T-cell depletion, it has great potential to serve as a marker of resistance to CAR-T therapy for the treatment of DLBCL. Furthermore, in a study utilizing single-cell RNA sequencing (scRNA-seq) with capture-based cell identification on anti-CD19 CAR-T cell infusion products, the molecular response at day 7 post-infusion was found to be significantly correlated with clinical response (*P* = 0.008). This suggests that the day 7 molecular response may serve as an early predictor of CAR-T cell efficacy [53].

#### 3.2.2 Primary Drug Resistance

In addition to markers of acquired resistance, T cells are also of interest in primary resistance [93]. For example, the function and potency of T cells before and after CAR-T infusion in the treatment of DLBCL also significantly impact drug resistance. In one study, primary resistance associated with T-cell dysfunction was assessed by CAR transgene expansion (Cmax), persistence (Tlast), impaired CD19+ B-cell regeneration, and initial response after CAR-T cell infusion, all of which were found to be correlated [98]. It has also been shown that greater multifunctionality, enhanced stemness, and reduced exhaustion of pre-infusion CAR-T cells correlate with improved disease control and increased CD19-CAR-T cell potency [99-101]. A study by Wang *et al.* demonstrated that the genetic features of T-cell immunodeficiency may be used to explain the mechanism of resistance to CAR-T therapy for DLBCL. It was hypothesized that in patients with innate immunodeficiencies, autologous CAR-T cells may undergo expansion and persistence impairments that reduce the effectiveness of CAR-T cells and lead to a poor prognosis [102]. Focusing on the adaptive and functional status of infused T cells during treatment is also a good way to predict drug resistance. Predictive models of T-cell subset proportions have been developed to predict clinical outcomes. A single-center retrospective study by Professor Zhao's team found that baseline T-cell subset proportions, collected prior to infusion, were positively correlated with early therapeutic response in patients with r/rNHL. The model used the ratio of central memory T cells (Tcm) to naïve T cells (Tn) within cytotoxic T cells (Tc) as a predictive biomarker, demonstrating high sensitivity and specificity [103]. Similarly, monitoring the proportion of T-cell subsets in drug-resistant relapse could be a possible predictive model.

### 3.3 Markers of Gene Expression

CAR-T cell treatment of DLBDL involves multiple genes and related pathways. Evaluation of circulating tumor DNA (ctDNA) in patients early in the course of CAR-T cell therapy has been shown to be predictive of progression, particularly in terms of disease recurrence, in a study monitoring early relapse in LBCL patients following infusion of axicabtagene ciloleucel [104]. Drug resistance is a significant cause of disease relapse. Therefore, one possible means of predicting resistance in CAR-T-treated DLBCL is to focus on circulating tumor DNA levels to monitor the occurrence of drug resistance. In addition to ctDNA, several related genes may serve as potential predictive markers of drug resistance. In a recent study, researchers analyzed data from more than 700 patients treated with CAR-T therapy and found that genetic alterations linked to drug resistance involved genes related to B cell identity (PAX5, IRF8), immune checkpoints (CD274), and the tumor microenvironment (TMEM30A) [105]. Additionally, research on the genes involved in regulating apoptosis in tumor cells is a significant area of study. For example, BCL2, a common anti-apoptotic gene, has been shown to be overexpressed and associated with drug resistance in DLBCL patients treated with R-CHOP [106]. It also serves as one of the tumor-intrinsic mechanisms of CAR-T resistance. Such a gene has great potential to serve as a predictive marker for drug resistance in CAR-T cell-treated DLBCL. Genes related to cell cycle regulation and DNA repair may also be one aspect of resistance predictors to be studied. For example, some researchers have found that cells resistant to CAR-T are depleted of genes that activate the cell death pathway, particularly the FADD, BID, CASP8, and TNFRSF10B [51]. TP53 has been suggested as a possible predictor of drug resistance. Analysis of the DLBCL genomic dataset in the study by Dufva *et al.* revealed that TNFRSF10B expression was negatively correlated with TP53 mutations. This heterogeneity in death receptor signaling may directly contribute to the emergence of primary resistance to CAR-T cell therapy in clinical care [107]. A number of prognostic mutational signatures (PMS) have also been identified using high-throughput genetic methods that may serve as predictors of long-term survival in DLBCL patients [108]. Focusing on PMS-associated molecular genes could also provide a possibility for predictive markers. Furthermore, abnormalities in the KMT2A gene are closely associated with the development of hematopoietic malignancies. In some patients with KMT2A rearrangements, CAR-T therapy is less effective, and relapses are concerning. It is even suggested that these relapses may represent a lineage switch during the progression of the disease [109]. Monitoring KMT2A gene alterations in CAR-T therapy is also a promising approach for understanding and managing resistance in DLBCL.

### 3.4 Markers of Tumor Microenvironment

The tumor microenvironment is a determinant of CAR-T cell efficacy in DLBCL. In previous studies, the prognostic and predictive roles of TME have been demonstrated in solid tumors, as well as the use of checkpoint inhibitors; however, there has been a lack of evidence on the predictive role of drug resistance in CAR-T cell therapy for DLBCL [110, 111]. However, a recent study found that CAR-T cells are markedly less effective in DLBCL patients whose tumor microenvironment displays features of T-cell exhaustion. It was found that inhibiting TGF-β in LBCL may reduce CAFs and enhance T-cell infiltration [41]. Through comprehensive evaluation, TGF-β in the tumor microenvironment may represent an emerging biomarker of therapy resistance. It is believed that the various features of TME and the interaction of its components with lymphoma cells demonstrate significant heterogeneity [112]. For example, in a study on intra-tumor heterogeneity in lymph node B-cell lymphomas, four transcriptionally distinct T-cell subpopulations were found to vary greatly in frequency in TME in samples from patients with DLBCL, suggesting that DLBCL can shape the microenvironment by regulating the recruitment of different T-cell subsets [113]. This represents a promising predictive approach; immunotherapy resistance can be anticipated by assessing the extent of T-cell infiltration in TME.

Cancer cells are usually surrounded by a complex tumor microenvironment. Previously, it has been shown that CAR-T cell therapy outcomes are correlated with TME immune content. Elevated concentrations of specific cytokines and chemokines, along with active cytotoxic T-cells and tumor-infiltrating lymphocytes in the tumor microenvironment before LBCL therapy, correlated with improved clinical results [114]. It has even been suggested that the immune cell composition in the tumor microenvironment predicts the prognosis of DLBCL. In one study, the expression of 730 immune-related genes was analyzed in tumor tissues from patients with DLBCL, and multiplexed immunohistochemistry was used to characterize T-cell phenotypes. Immunophenotypic analysis of different cellular subpopulations showed that a high proportion of immune-checkpoint-positive T cells is associated with a poorer survival rate [115]. Drug resistance has also been studied as a prognostic factor, and aspects of the immune cell composition of the tumor microenvironment have the potential to impact the prediction of drug resistance in CAR-T cell treatment of DLBCL. The prognostic significance of serum LDH levels as an excellent surrogate marker of tumor load in DLBCL patients has been demonstrated in previous studies [116]. In contrast, LDH and increased tumor volume are also potent adverse risk markers in patients treated with CAR-T cells [117, 118]. Additionally, tumor load is associated with CAR-T resistance [119, 120]. The serum LDH level, as an easily measurable biomarker, may be helpful in predicting resistance to CAR-T cell therapy in DLBCL patients. Furthermore, glycolysis has been implicated in the regulation of TME [121]. A recent retrospective study demonstrated that certain markers of glycolytic biology are correlated with the prognosis of DLBCL patients [122]. However, there are no reliable glycolytic biomarkers for predicting drug resistance in DLBCL patients treated with CAR-T. It is expected that, in the future, reliable markers will be discovered for predicting drug resistance and facilitating patient treatment (Fig. **1**).

## 4 NEW STRATEGIES TO OVERCOME DRUG RESISTANCE

In recent years, researchers have implemented a series of improvements to address the issue of drug resistance in DLBCL treated with CAR-T, achieving promising results (Table **1**). A commonly used approach is to design CAR molecules that can target two or more antigens on tumor cells simultaneously, thereby overcoming antigen loss after CAR-T cell therapy [123]. For example, CAR-T cells that simultaneously target both CD19 and CD22 have been engineered to effectively combat relapsed or refractory B-cell cancers [76]. This approach has yielded promising results and is a viable modality in B-ALL and is believed to hold considerable promise in DLBCL [124]. In addition, combinations of drugs can further overcome drug resistance. For example, the combination of CAR-T and anti-PD1 is utilized to overcome resistance mechanisms and improve efficacy. There are currently promising clinical results using the CD19/22 dual-targeting CAR-T (AUTO3) when combined with pembrolizumab for patients with relapsed or refractory LBCL, showing durable remission for more than 12 months in 54.4% of those in complete remission [125]. As technology for gene editing advances, it holds great promise in overcoming CAR-T resistance. There have been reports of using CRISPR/Cas9 to edit multiple genes in allogeneic CAR-T cells. Findings have shown that CRISPR/Cas9 editing for CAR expression can be a secure and effective approach, and it is possible to target CAR to the TCR in the T-cell's TRAC site [126, 127]. Overall, gene editing techniques offer new approaches to overcoming drug resistance in DLBCL. There have also been studies on modifying CAR-T cells to improve efficacy in targeting the mechanism of T-cell depletion that leads to drug resistance. These modified CAR-T cells can enhance their cytotoxic effect by releasing cytotoxic proteins or activating immune cells [128]. For example, T cells engineered as redirected for generalized cytokine-mediated killing (TRUCKs) demonstrate improved anti-tumor effects. Some TRUCKs secrete IL-12, which has shown promising results in mouse models and is believed to be relevant for future clinical studies [123, 129].

## CONCLUSION AND FUTURE PROSPECTS

In recent years, CAR-T cell therapy has revolutionized the treatment landscape for DLBCL, yielding unprecedented therapeutic response rates and demonstrating a highly promising therapeutic approach. However, CAR-T cell resistance remains a major challenge to the long-term and far-reaching success of this therapeutic approach. In the future, the study of drug resistance markers for DLBCL will be a key direction for CAR-T cell therapy. Through in-depth research on drug resistance-related markers, we can understand the principles of drug resistance mechanisms and explore new therapeutic targets and drugs. Current studies have demonstrated that the mechanism of drug resistance in DLBCL cells involves a range of factors, including alterations in cell surface receptors, aberrant activation of signaling pathways, and immune evasion. Predictive markers of drug resistance designed to address the relevant resistance mechanisms include surface receptor markers, T-cell depletion markers, and TME markers.

It has been observed that there are few predictive markers for drug resistance in DLBCL; however, several predictive markers exist for related blood diseases. For instance, TP53 mutations, FLT3-ITD-driven signaling pathway aberrations, and CXCR4/CXCL12-mediated bone marrow niche protection in AML collectively suggest that genomic instability and microenvironmental remodeling may represent universal mechanisms underlying multidisease resistance. Similarly, in multiple myeloma (MM), B-cell maturation antigen (BCMA) downregulation and clonal heterogeneity-driven therapeutic evasion highlight mechanistic overlaps with antigen loss and subclonal evolution observed in DLBCL. Furthermore, BTK- and BCL-2 mutation-mediated resistance to targeted therapies in chronic lymphocytic leukemia (CLL) highlights the potential for predicting CAR-T efficacy through dynamic monitoring of genetic alterations and metabolic reprogramming. Future investigations should integrate cross-disease datasets to explore the applicability of predictive biomarkers and resistance mechanisms identified in these hematologic malignancies for DLBCL. Validating such analogies *via* multiomics profiling (*e.g.*, epigenomic-metabolomic crosstalk) and preclinical models (*e.g.*, patient-derived xenografts with microenvironmental reconstitution) could unveil conserved resistance signatures. This approach will facilitate optimization of predictive frameworks and the design of combinatorial therapeutic strategies, such as coupling CAR-T with microenvironment-targeting agents or epigenetic modulators to preempt resistance. Systematic validation through multi-institutional cohorts will be crucial to establishing the generalizability of these cross-disease insights for the precision management of refractory DLBCL.

Although definitive research and clinical studies on these markers are still lacking in this area, we are optimistic that in the future, more accurate and effective predictive markers will be used in the treatment of DLBCL with CAR-T.

## AUTHORS' CONTRIBUTIONS

The authors confirm their contribution to the paper as follows: Conceptualization: XMT, YW, and JQY; data curation: SQY, LLC, and LX; formal analysis: QJF and YZL; writing the original draft: SQY; writing, reviewing, and editing: SQY, JQY, and YYZ; visualization: SQY and MXJ.

## Figures and Tables

**Fig. (1):**
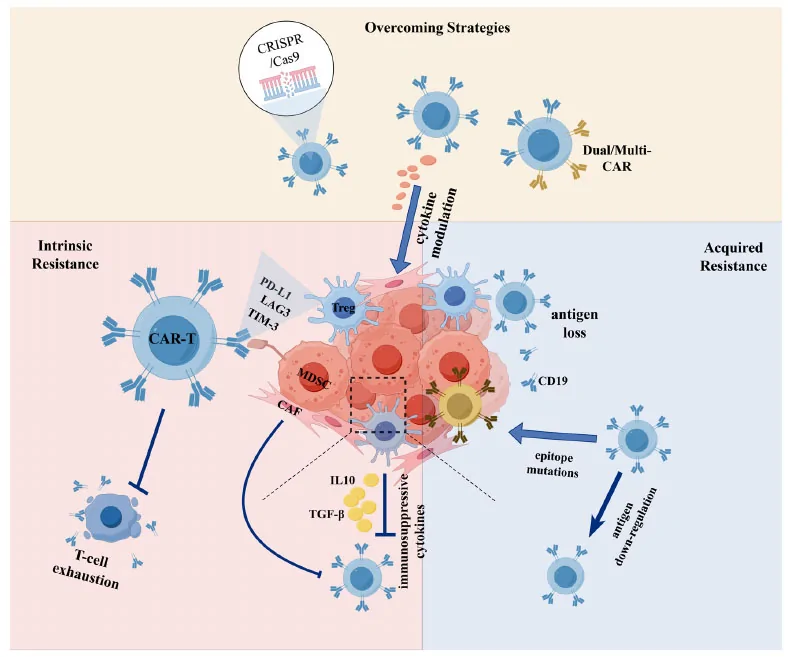
Mechanisms of resistance and overcoming strategies. Mechanisms of resistance include intrinsic resistance, tumor microenvironment features (*e.g*., immune checkpoint molecules, Tregs, and MDSCs), and defective CAR-T cell function (*e.g*., T-cell exhaustion). Acquired resistance in volves antigen escape mechanisms (*e.g.,* CD19 loss, epitope mutations, antigen down-regulation). Overcoming strategies are mostly based on engineering approaches (e.g., dual-target CARs, cytokine modulation, CRISPR/Cas9-mediated editing of CAR-T cells' genes).

**Table 1:** Clinical trials targeting CAR-T for B-cell lymphoma in the last five years1.

**Main ID**	**Countries of Recruitment**	**Public Title**
NCT06209619	United States	CC-99282 + Rituximab Early Post CART for Non-Hodgkin's Lymphoma [130]
NCT06247501	Republic of Korea	Treatment of CD19 Chimeric Antigen Receptor T Cells for Pediatric Patients with CD19-positive B-cell Acute Lymphoblastic Leukemia Who Are Indicated for Hematopoietic Stem Cell Transplantation [131]
NCT06160362	China	The Safety and Efficacy of Double-target CART-19 and 20 Cells in Non-Hodgkin's Lymphoma (NHL) [132]
NCT06101381	Brazil	CD19-directed CAR-T Cell Therapy for R/R Acute Leukemia and Lymphoma CARTHEDRALL [133]
ChiCTR2300075912	China	HD CD19 CAR-T Therapy in Patients with Refractory or Relapsed Aggressive B-cell Non-Hodgkin Lymphoma: A Phase Ib/II Clinical Trial [134]
NCT05514327	China	A Study of Ultra-fraction Radiotherapy Bridging CART in R/R DLBCL [135]
NCT04381741	China	CD19 CAR-T Expressing IL7 and CCL19 Combined With PD1 mAb for Relapsed or Refractory Diffuse Large B Cell Lymphoma CICPD [136]
NCT05239676	China	Autologous Hematopoietic Stem Cell Transplantation Combined with CAR-T Cells in the Treatment of Refractory and Relapsed Malignant Lymphoma [137]
TCTR20210818003	Thailand	Safety/Phase I Study of PiggyBac Transposon-Mediated Chimeric Antigen Receptor T Cells Targeting CD-19 in Thai Patients with Relapsed or Refractory B-cell Acute Lymphoblastic Leukemia and Lymphoma [138]
NCT04007029	United States	Modified Immune Cells (CD19/CD20 CAR-T Cells) in Treating Patients with Recurrent or Refractory B-Cell Lymphoma or Chronic Lymphocytic Leukemia [139]
NCT03876028	United States	Study of Tisagenlecleucel in Combination With Ibrutinib in R/R Diffuse Large B-cell Lymphoma Patients [140]
NCT05665062	United States	Autologous CD19 CAR-T Cell Therapy (SYNCAR-001) + Orthogonal IL-2 (STK-009) in Subjects with CD19+ Hematologic Malignancies [141]
NCT06365671	United States	CAR-T Following ASCT for Relapsed/Refractory B-Cell Non-Hodgkin's Lymphoma (R/R B-NHL) with High-Risk Prognostic Factors [142]
ChiCTR2100052798	China	Efficacy Observation of Chidamide Combined with PD-1 Monoclonal Antibody after CART for Relapsed/Refractory B-cell Lymphoma—A Prospective, Single-Center, Single-Arm Clinical Study [143]
NCT04257578	United States	Acalabrutinib and Anti-CD19 CAR T-cell Therapy for the Treatment of B-cell Lymphoma [144]
NCT04231747	United States	A Study of CC-97540, CD19-targeted NEX-T Chimeric Antigen Receptor (CAR) T Cells in Subjects with Relapsed or Refractory B-cell Non-Hodgkin Lymphoma [145]

**Note:** Data cited in WHO ICTRP.

## LIST OF ABBREVIATIONS

ALL: Acute Lymphoblastic Leukemia
BCMA: B-cell Maturation Antigen
CAFs: Cancer-associated Fibroblasts
CAR: Chimeric Antigen Receptor
CLL: Chronic Lymphocytic Leukemia
CR: Complete Remission
ctDNA: Circulating Tumor DNA
DLBCL: Diffuse Large B-cell Lymphoma
ECM: Extracellular Matrix
FDA: Food and Drug Administration
Foxp3: Forkhead Box P3
HDC/ASCT: High-dose Chemotherapy With Autologous Stem Cell Transplantation
IL-10: Interleukin-10
IL-12: Interleukin-12
IL-7: Interleukin-7
IRF8: Interferon Regulatory Factor 8
LAG3: Lymphocyte-Activation Gene 3
LBCL: Large B-cell Lymphoma
MCP-1: Monocyte Chemoattractant Protein-1
MDSCs: Myeloid-Derived Suppressor Cells
MM: Multiple Myeloma
NHL: Non-Hodgkin Lymphoma
ORR: Overall Response Rate
OS: Overall Survival
PAX5: Paired Box Protein 5
PD-1: Programmed Cell Death Protein 1
PD-L1: Programmed Death-Ligand 1
PMS: Prognostic Mutational Signatures
Pola-R-CHP: Polatuzumab Vedotin in Combination with Rituximab, Cyclophosphamide, doxorubicin, and prednisone
R-CHOP: Rituximab Combined with Cyclophosphamide, Doxorubicin, Vincristine, and Prednisone
R/R: Refractory/Relapsed
scFv: Single-chain Variable Fragment
TAAs: Tumor-Associated Antigens
TCR-CD3: T-cell Receptor-CD3 Complex
TGF-β: Transforming Growth Factor β
TIGIT: T Cell Immunoreceptor with Immunoglobulin and Tyrosine-based Inhibitory motif (ITIM) domain
TIM-3: T-cell Immunoglobulin and mucin-domain Containing-3
TME: Tumor Microenvironment
TMEM30A: Transmembrane Protein 30A
TNFRSF10B: Tumor Necrosis Factor Receptor Superfamily Member 10B
TNFRSF14: Tumor Necrosis Factor Receptor Superfamily Member 14
Tregs: Regulatory T Cells
TSAs: Tumor-Specific Antigens
